# Is the degree of clonality of forest herbs dependent on gap age? Using fingerprinting approaches to assess optimum successional stages for montane forest herbs

**DOI:** 10.1002/ece3.23

**Published:** 2011-11

**Authors:** Kathrin Patsias, Helge Bruelheide

**Affiliations:** Institute of Biology/Geobotany and Botanical Garden, Martin Luther University Halle WittenbergAm Kirchtor 1, 06108 Halle (Saale), Germany

**Keywords:** AFLP, *Calamagrostis villosa*, Clone sizes, Gap dynamics, *Trientalis europaea*, *Vaccinium myrtillus*

## Abstract

Using molecular fingerprinting (amplified fragment length polymorphism [AFLP] method), we explored the potential of small-scale population analysis for understanding colonization patterns of herb layer species in forests after canopy disturbance. We investigated three common forest understorey species with different life forms (*Trientalis europaea, Calamagrostis villosa*, and *Vaccinium myrtillus*) in the Harz Mountains in Germany in three different gap age classes and undisturbed forest. For two of them (*T. europaea* and *C. villosa*), we analyzed clone sizes and clonal structure. We hypothesized that clone sizes depend on age since gap formation and are affected by light availability. Mean patch sizes of *V. myrtillus, T. europaea*, and *C. villosa* formed were 3.7 m^2^, 27.9 m^2^, and 40.6 m^2^, respectively. *Trientalis europaea* and *C. villosa* patches consisted mostly of more than one genet. Largest clone sizes of *T. europaea* were encountered in gaps of intermediate successional age (15–60 years, averaged minimum estimation of clone sizes: 6.56 m^2^) whereas clone size of *C. villosa* was found to be independent from gap age and had a mean minimum clone size of 0.49 m^2^. In both species, clone size was positively related to light availability. Additionally, there was a positive relationship between clone size and ramet density for *T. europaea* and *C. villosa.* Genetic variation was higher within populations of *T. europaea* and *C. villosa* than among populations. *Trientalis europaea* was the only species with a clear genetic isolation by distance, pointing at an equilibrium between gene flow and genetic drift. In conclusion, we showed that forest canopy gap dynamics clearly affect the small-scale structure of populations of understorey plants. Species with high lateral growth rates, such as *T. europaea* offer the possibility to serve as “ecological clock” for dating ecological processes.

## Introduction

Molecular fingerprinting techniques have been successfully applied to analyze plant colonization events at various spatio-temporal scales, ranging from dispersal in historical biogeography (e.g., Westergaard et al. 2011) to global invasion routes (e.g., Lachmuth et al. 2010). Much less attention has been paid to small-scale colonization patterns that are shaped by vegetative growth and sexual reproduction (Eriksson 1989). In a recent review, Zobel et al. (2010) concluded that at small scales little is known about the role of clonality in generating vegetation patterns.

This is particularly true for forest vegetation, although most understorey herb layer species are characterized by a preponderance of clonal growth compared to sexual reproduction. Of all species in the central European flora, 66. 5% are clonal (Klimeš et al. 1997). However, only a few studies investigated clone sizes of species as a function of their environment. For *Pteridum aquilinum*, Oinonen (1967) described a clear dependence of clone size on the time elapsed since a distinct disturbance event (e.g., fire) because the establishment of new populations is only possible on open ground. Knowing the age of distinct fire events, Oinonen (1967) could assess exact clone ages. Furthermore, the clone age was shown to be proxy for clone size (Oinonen 1969). The older the clones, the larger their size, a pattern that also holds for other life forms as grasses, *Calamagrostis epigejos*, or herbs, *Convallaria majalis* (Oinonen 1969), and even for trees (Torimaru and Tomaru 2005; Vonlanthen et al. 2010). This size–age relationship is caused by genets that spread laterally by growing increasing numbers of ramets with time (Hartnett and Bazzaz 1985). In their study on the clonal growth of seagrass, Ruggiero et al. (2005) have pointed out that molecular approaches to analyze the clonal structure of plants have to consider that patches might not only grow from single genets but that genets might establish themselves by clonal growth repeatedly in already existing clones (initial vs. repeated seedling recruitment sensu Eriksson 1989, 1993). Similarly, the type of clonal growth will determine the resulting patterns, with a mosaic structure arising from a phalanx strategy, where genetically identical ramets are clustered and discernable as discrete units, or an intermixture of genets in a guerrilla strategy (Lovett–Doust 1981). While spatial autocorrelation analysis might give insight into the differential contribution of sexual and vegetative recruitment, actual clone sizes can only be detected by explicit spatial mapping (Vonlanthen et al. 2010).

Another difficulty is that all fingerprinting methods are error-prone. In particular, some marker systems are not well reproducible, such as RAPD (randomly amplified polymorphic DNA) or ISSR (inter simple sequence repeats), compared to AFLP (amplified fragment length polymorphism) with a much higher degree of reproducibility (Jones et al. 1997).

An obvious determinant of clonal structure of forest plants is disturbance, resulting in canopy gap creation and subsequent gap closure. Herb layer species strongly respond to the ensuing changed environmental conditions, in particular to a higher light availability compared to undisturbed forest matrix (Canham et al. 1990). However, the habitat suitability often takes the form of a temporal optimum curve. Directly after gap creation, understorey species adapted to shade often suffer from a too high irradiance and too low soil moisture (Mitamura et al. 2009). With gap development, populations can expand as long as competition intensity is low (Hughes and Fahey 1991), then reach a maximum size until light availability decreases and competition increases in the course of gap closure (Widmer 1998). From an ecological point of view it would be interesting to see how much the temporal course of habitat suitability is congruent across different life forms. Such comparisons have not been made so far with fingerprinting studies on clonal growth, as most studies have only focused on singular species (Arens et al. 2005; Wilson et al. 2005; Jacquemyn et al. 2006). It can be assumed that the species-specific clone growth will depend on relative growth rates, which in turn strongly depend on life form (Grime and Hunt 1975). Thus, dwarf shrubs might reach maximum clone sizes in later successional stages compared to herbaceous or pseudoannual species, simply because they grow more slowly. For this reason, we included three different life forms in our studies, which were among the most abundant species in the near natural spruce forest in the Harz Mountains in Central Germany. The fast growing herb (*Trientalis europaea*) and grass species (*Calamagrostis villosa*) produce long stolons and rhizomes, respectively, and consequently, can be described as guerrilla strategists (Pyšek 1993 and references within; Taylor et al. 2002). In contrast, the slow growing dwarf shrub (*Vaccinium myrtillus*) is representative of the phalanx strategy (Albert et al. 2004). Thomas and Hay (2008) studied seven clonal herbs and found hints for differences also within the guerrilla group. For example, both *Trifolium repens* and *Glechoma hederacea* are considered guerrilla strategists but they are clearly separated regarding their morphological and physical traits (Thomas and Hay 2008). The present study followed this finding by focusing on differences in clone size and clonal structure in two species of the same strategy, i. e. the guerrilla strategy.

Another aspect of clonal growth is the temporal development of ramet density. Assuming an anisotropic growth of clones, ramets of a clone will be produced both outwards and inwards. Consequentially, it can be expected that ramet density, to a certain extent, might also increase with time, even if the ramet longevity would be short. Thus, there should be a positive relationship between ramet density and clone size. Furthermore, older patches have adjacent to a higher ramet density also a higher genetic diversity than young patches (Scheepens et al. 2007). In contrast, when competition between genotypes is high, one genotype might outcompete others, and therefore, old patches would still have higher ramet density but lower genetic diversity than young patches (Gray 1987; Silvertown 2008).

In particular, we hypothesized: (1) There is a unimodal relationship between clone size and gap age with optimum in mid-successional aged gaps. In addition, we also tested whether clone sizes increase with light availability. (2) Fast growing species attain larger clone sizes and reach maximum clone sizes at earlier successional stages in gaps. (3) Ramet density is positively related to clone size. (4) Assuming a balance of gene flow and random genetic drift, we hypothesized that there is isolation by distance across all populations.

## Methods

### Study area and target species

We investigated three species (two of them with molecular fingerprinting) with different life forms, the herb *T. europaea* L. (Primulaceae), the grass *C. villosa* (CHAIX) J. F. GMEL. (Poaceae), and the dwarf shrub *V. myrtillus* L. (Ericaceae). These three species are the major players in the species poor spruce forest on Mt. Brocken (51°48′02″N, 10°37′02 ″E, 1,142 m a.sl.), the highest elevation of the Harz Mountains, Central Germany (for a species list of the study area see Kirchner et al. 2011). The forest on Mt. Brocken is protected from human impact since 1937 and can be considered one of the very few forest stands in Germany with characteristics of a primary forest. Steep slopes and surrounding mires made these eastern slopes of Mt. Brocken virtually inaccessible, confining timber removal to only a few and locally restricted areas. Forest management has never taken place (Stöcker 2002). Disturbance agents resulting in canopy gaps in this near-natural forest are mainly wind, single tree fall, and bark beetle infestations (Wegener et al. 2003). The herb layer usually remains partially intact after such disturbance events, and thus, gaps closure occurs by remaining herb and tree individuals as well as by new colonization via seeds or by clonal growth from the edge. Another feature of this forest is its openness, allowing for a moderate but continuous regeneration of spruce also in undisturbed sections (Kathke and Bruelheide 2010a).

*Trientalis europaea* is a pseudoannual small herb, which reaches a height of approximately 15 cm and prefers clonal growth over sexual reproduction (Hiirsalmi 1969; Kirchner et al. 2009). Since *T. europaea* follows the initial seedling recruitment strategy, this species depends mostly on disturbance for seedling recruitment and develops later by clonal propagation (Eriksson 1997). A high mobility has been described for *T. europaea* (Piqueras and Klimeš 1998), which is typical of the guerrilla strategy. Piqueras and Klimeš (1998) showed that *T. europaea* produce stolons that can reach up to almost 1 m but have a mean stolon length of 17.2 cm. In a previous study, we calculated a minimum mean stolon length of 5.4 cm and a mean density of 20 ramets per m^2^ for this species on Mt. Brocken (Kirchner, unpubl. data).

*Calamagrostis villosa* also displays a guerrilla strategy, growing with long rhizomes (Prach and Pyšek 1994) and a dense root system (Betz 1998). Additionally, also *C. villosa* shows predominantly vegetative compared to sexual reproduction (Pyšek 1993). Since most of the caryopses are sterile, the germination rate of *C. villosa* is less than 10% (Pyšek 1993).

In contrast to the other two target species, *V. myrtillus* clones expand by the phalanx strategy (Albert et al. 2004), forming dense patches with a mean annual growth rate of rhizomes of approximately 7 cm (Flower–Ellis 1971). *Vaccinium myrtillus* is a deciduous dwarf shrub with preference of vegetative reproduction as seedling establishment is inhibited in the dense patches (Ritchie 1956).

### Sampling design and genetic analyses

**Figure 1:**
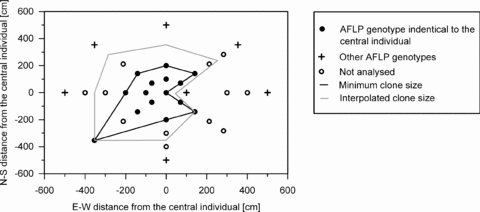
Sampling scheme for a *T. europaea* patch and the results of the AFLP analyses. The black line gives the minimum clone size of the central clone and comprises all samples with the same AFLP genotype (black dots). The gray line encloses the area of the interpolated clone size of the central clone (see Methods for further details). The crosses indicate samples with genotypes different from that of the central individual and open circles show samples that were taken in the patch but have not been analyzed.

**Figure A1:**
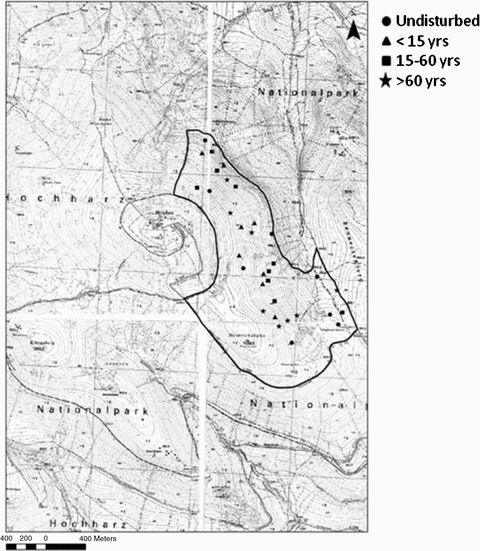
Distribution of the study plots in the study area according to four gap age classes (undisturbed: dots, <15 years: triangles, 15–60 years: squares, >60 years: asterisks).

We established 40 study plots (10 × 10 m^2^) randomly in the study area (size: 225 ha, see Fig. A1), stratified by four gap age classes (undisturbed forest: 10 plots, <15 years: 10 plots, 15–60 years: 10 plots, >60 years: 10 plots). The gap age was estimated from high-quality aerial photographs (taken in 1945, 1991, 2000, and 2003) and was confirmed by taking tree cores and dendrological analyses (for more details see Kathke and Bruelheide 2010a, b). Gap sizes ranged from 344 m^2^ (gaps smaller than 100 m^2^ were neglected) to 12,902 m^2^ with an average of 3,327 m^2^. The distance between the study plots was on average 961 m (minimum distance between two plots: 60 m, maximum distance between two plots: 2,560 m). Every species was analyzed in every plot. One patch (i.e., population) of every target species was selected that grew nearest to the center of the plot. As the plots were placed strictly randomly and not deliberately located in the center of a gap, the selected patches could have any position within a gap. The center of the patch of one of the target species was marked and shoots were sampled on five positions in the eight cardinal compass directions, covering the visible extension of that patch (Fig. 1). The distance between samples depended on patch size and was chosen in a way to divide the radius of the patch in five equal sections. This resulted in a potential total size of 41 (eight times five for the eight arms of the sampling star and one sample in the center). However, in most cases sampled ramet number was lower because of irregular patch sizes. In addition, the expansion of the patch was measured along these eight directions and the density of shoots per m^2^ was assessed. The sampled tissues were put immediately on silica gel. Finally, we measured light availability as relative photosynthetic photon flux density (PPFD) in a 2 × 2 m^2^ grid at the study plots, resulting in 36 measurement points. Relative light availability of a patch was obtained by averaging the PPFD values of the four measurement points closest to the patch center (see Kirchner et al. 2011 for details).

Clone size estimation followed stepwise procedure, designed in a way to successively improve the identification of the clone's boundary by iteratively including points situated between samples with differing AFLP signatures. The procedure is explained in detail in Vonlanthen et al. (2010). In first step, we analyzed a subset of samples for *T. europaea* and *C. villosa* (the central individual and the closest individual in each of the eight compass directions, resulting in maximum nine samples per patch, called clone ring 1). This step revealed whether any clonality could be expected at all. In total, we analyzed in this first step 226 and 299 samples for *T. europaea* and *C. villosa*, respectively. To estimate the clonal diversity, we calculated the clonal richness (*R*) after the formula of Ellstrand and Roose (1987) as the number of detected clones (*G*) divided by the sample size (*N*):

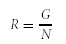


As the mean clonal richness was significantly higher for *C. villosa* than for *T. europaea* in this first investigation step (*R*_*C. villosa*_ = 0.67 and *R _T. europaea_* = 0.44), the second step in the analysis differed between both species. In both species, we analyzed the individuals adjacent to the already investigated samples further to the periphery. In addition, in *T. europaea*, the most peripheral individuals in each compass direction were included. Thus, in total, we analyzed 529 and 571 samples for *T. europaea* and *C. villosa*, respectively. Initially, a third step was planned to analyze more samples between the central and most peripheral ramets, but this turned out to be unnecessary as in the majority of samples in *C. villosa* clone boundaries were encountered already within the central samples and in *T. europaea* many of the most peripheral samples turned out to have the same genotype as the central one. However, this nested approach of iteratively improving the estimate of clone boundaries is equally applicable for clone sizes of less than a square meter to the scale of square kilometers (Vonlanthen et al. 2010).

For the DNA extraction, we used the ATMAB (Alkyltrimethylammonium bromide) protocol after Dumolin et al. (1995) for *T. europaea* and *C. villosa*. As this method failed for *V. myrtillus*, there are only data on patch size and density, but no fingerprinting results for this species.

After measuring the DNA concentration in the samples with the NanoDrop 1000 spectrometer (PEQLAB Biotechnologie GmbH, Erlangen, Germany), we conducted the AFLP method after Mannschreck et al. (2002), with only a few minor modifications. In the first AFLP-step (the restriction and ligation), we incubated the samples over night under room temperature. The preamplification had the following PCR conditions: 5 min at 95°C and 25 cycles of 20 sec at 94°C, 35 sec at 56°C and 2 min at 72°C, and finally 30 min at 62°C. In the main amplification, the PCR protocol was: 2 min at 94°C, 10 cycles of 20 sec at 94°C, 30 sec at 66°C, with lowering the temperature for 1°C in every cycle, and 2 min 72°C. Afterwards, 20 cycles with 20 sec at 94°C, 30 sec at 56°C, and 2 min at 72°C were passed, and finally 30 min at 60°C.

We used three primer triples for *T. europaea* (Mse1-CAT/EcoR1-ACT [FAM]/-AAC [HEX], Mse1-CTC/EcoR1-AAG [FAM]/-AAC [HEX], and Mse1-CTA/EcoR1-AAG [FAM]/-AAC [HEX]). For *C. villosa*, we used three primer pairs (Mse1-CAG/EcoR1-AAG [FAM], Mse1-CAG/EcoR1-AGA [FAM], and Mse1-CAG/EcoR1-ACT [FAM]). These products were analyzed with MegaBACE 1000 (Amersham Biosciences, Freiburg, Germany). The reaction mix for the sequencer contained 2 μl of the product of the main amplification, 0.3 μl ET400-R size standard (GE Healthcare Europe GmbH, Freiburg, Germany), and 5.6 μl H_2_O. We used the GE Healthcare protocol for genotyping.

### Data analyses

The Fragment Profiler (version 1.2, by Amersham Biosciences, 2003) was used to create a 0/1 matrix of all polymorphic loci, whereas 0 means absence and 1 presence of a peak on the respective locus. For the final analyses, we scored only loci that were clearly detectable (total number of scored loci for *T. europaea* and *C. villosa* were 187 and 131, respectively).

Following the procedure outlined in Vonlanthen et al. (2010), we calculated a threshold for the two target species on the basis of doubly analyzed samples (48 and 49 samples for *T. europaea* and *C. villosa*, respectively), using a Sørensen Index of 4.71% for *T. europaea* and 2.34% for *C. villosa*. This threshold avoids the type 1 error described in the study of Schnittler and Eusemann (2010). Schnittler and Eusemann (2010) described the consequences of genotyping errors for clonality estimation and define the type 2 error as splitting the same clone into different genotypes, because of failing to reliably reproduce the same fingerprints. As these thresholds are comparably low, our clone size estimates are conservative. For assigning samples to the central clone, we used cluster analyses based on Sørensen differences and the single linkage algorithm, as implemented in R 2.11.0 (R Development Core Team 2010).

On the basis of the geographical distances between two samples, we calculated the clone sizes of the second investigation step, which were approximated by polygons (see Fig. 1). As not all samples that were taken in the field were analyzed with AFLP, we defined a minimum clone size as well as an interpolated clone size. Both size measures referred to the clone that included the central ramet of the sampled patch. The calculation of the minimum clone size was based only on those samples that had the same AFLP-genotype as the central individual, placing the enclosing polygon exactly through the position of these samples. For the interpolated clone size, we assumed that the limit of the clone was somewhere in between the most peripheral ascertained genotyped sample of that clone and the next peripheral ramet that had been assigned to a different genotype. Thus, the enclosing polygon was located halfway to the next ascertained genotype to the periphery, irrespective of further samples had been taken between that had not been analyzed (Fig. 1). If the genotyped sample of a clone was the most peripheral one analyzed, the polygon was not expanded beyond that point. This procedure only gave one clone size per patch. However, especially in the case of *C. villosa*, the central clone was not always the largest one. Thus, we also calculated the minimum clone size as described above for the largest clone that was encountered in a patch.

For analyzing the genetic structure, the data were compiled in two datasets; the first one including all genotyped samples (the ramet dataset) and the second one including each clone only once (the genet dataset). Analysis of molecular variance (AMOVA) was carried out with GenAlEx (version 6. 4, Peakall and Smouse 2006), assessing the variation among gap age classes and the variation among and within population variation, the Φ_PT_ and the Φ_RT_ values. Presence/absence of AFLP alleles was subjected to principle coordinate analysis (PCoA), and a Mantel-test was calculated for analyzing the relationship between Φ_PT_ and geographic distance, both using the R 2.11.0 software.

Patch sizes were analyzed with mixed effects models (proc mixed, SAS 9. 1, SAS Institute Inc. 2002), considering species and gap age class as fixed factors and plot (nested in gap age) as random factor. As clone sizes and ramet density had not been measured only once per plot, these response variables were analyzed with general linear models (proc glm), separately by species, with gap age as fixed factor. Additionally, we calculated a contrast between undisturbed and disturbed forest stand with the estimate and contrast statements in SAS.

Finally, we simulated the expansion rate of *T. europaea* and *C. villosa* as well as the clone age of *T. europaea*, making use of the mean annual expansion rate of 17.2 cm as provided by Piqueras and Klimeš (1998). The underlying null model was a random walk of shoots and stolons, in each time step assuming a random angle from a mother ramet to a daughter tuber of *T. europaea* but with a fixed distance of 17.2 cm. The distance to the starting position was calculated and the process was repeated till the observed clone sizes (calculated by AFLP analyses) were reached. Clone area was assumed to be circular with the resulting radius after a certain period. Averaging the resulting clone area over 1,000 random walks gave a sufficiently good estimate of the clone age–size relationship and allowed the estimation of clone age for *T. europaea*. We reversed the procedure for estimation of clone expansion rates from gap ages, using the same simulation model.

## Results

### Patch size

**Figure 2:**
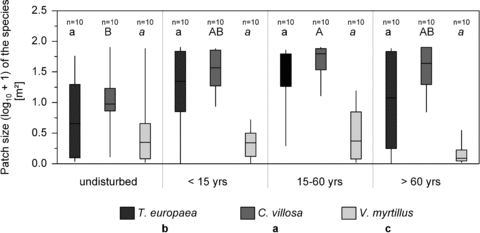
Boxplots (with median, upper and lower quartile, maximum and minimum) of patch size (log_10_+1 transformed) of the three target species (*T. europaea, C. villosa*, and *V. myrtillus*) in the four gap age classes (undisturbed, <15 years, 15–60 years, and >60 years). Bold letters in the legend show the result of the Tukey–Kramer post-hoc test between species. Letters in the graph indicate the results of the Tukey–Kramer post-hoc test between the gap age classes within the species (small letters: *T. europaea*, capital letters: *C. villosa*, and italic letters: *V. myrtillus*).

The most discrete patches were found for *V. myrtillus*, while individuals of the two other target species were more continuously distributed. *Calamagrostis villosa* formed the largest patches with on average 40.6 m^2^, with an intermediate size of 27.9 m^2^ in *T. europaea* and smallest (3.7 m^2^) in *V. myrtillus* (Fig. 2). Maximum patch sizes were quite similar for all three species, with 78.5 m^2^, 78.5 m^2^, and 74.6 m^2^ for *C. villosa, T. europaea*, and *V. myrtillus*, respectively. Patch size of *C. villosa* was significantly correlated with gap age (*F* = 4.25, *P* = 0.011), while no relationship was encountered for *T. europaea* and *V. myrtillus* (*F* = 2.14, *P* = 0.113 and *F* = 1.52, *P* = 0.226, respectively). *Calamagrostis villosa* showed largest patches in gaps between 15 and 60 years and the smallest in undisturbed forest, with a mean patch size of 54.14 m^2^ and 21.13 m^2^, respectively (Fig. 2). In contrast, there was a significantly positive relationship between gap size and patch size for *C. villosa* and *T. europaea* (*P* for the relationship between log_10_ [gap size + 1] and log_10_ [patch size + 1] was 0.008 and 0.021, respectively) and a negative trend for *V. myrtillus* (*P* for the relationship between log_10_ [gap size + 1] and log_10_ [patch size + 1] 0.099). Additionally, in all species, large patches were denser. We found a highly significant relationship (*P* < 0.001) between patch size and shoot density for all of our target species, (*r*^2^ for the relationship between log_10_ [patch size + 1] and the log_10_ [shoot density + 1] of 0.843, 0.817, and 0.723, for *T. europaea, C. villosa*, and *V. myrtillus*, respectively.

### Clone size

**Figure 3:**
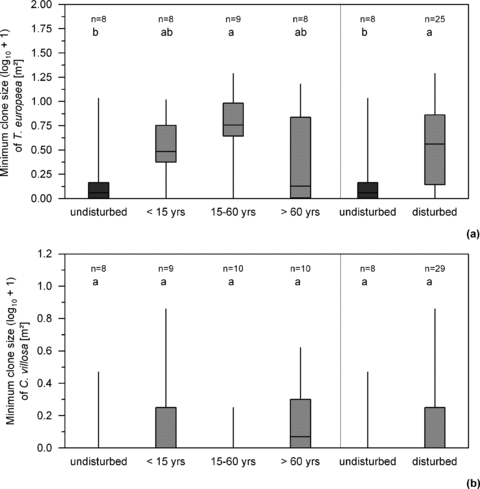
Boxplots (with median, upper and lower quartile, maximum and minimum) of clone size (log_10_+1 transformed) of the central clone of (a) *T. europaea* and (b) *C. villosa* in the four gap age classes (undisturbed, <15 years, 15–60 years, and >60 years). The right-hand side of the graphs shows the contrast between undisturbed versus gap plots. Small letters indicate statistically significant differences between the gap age classes according to the Tukey–Kramer post-hoc test.

In the first investigation step, the results of the clone ring 1 showed larger clone sizes for *T. europaea* than for *C. villosa* (*F* = 13.81, *P* = 0.001). For all investigated samples together, clone size showed a similar dependence on gap age as encountered for patch size. This trend was clearer for *T. europaea* than for *C. villosa* (Tables 1 and 2). In general, the central clone of *T. europaea* was larger in gaps than in undisturbed forest (Fig. 3A), with an averaged maximum size encountered in gaps between 15 and 60 years and a minimum size in undisturbed forest (mean minimum clone size of the central clone 6.56 m^2^ and 1.39 m^2^, respectively, Table A1 and Fig. 3A). In contrast, the central clones of *C. villosa* did not differ in size, neither between the several gap age classes nor between gaps and undisturbed forest stands (Fig. 3B; Table 2). Similarly, the interpolated clone sizes differed for *T. europaea* between disturbed and undisturbed forest (mean interpolated clone sizes 8.95 m^2^ and 3.28 m^2^ for disturbed and undisturbed forest, respectively, see Table 1), but not for *C. villosa* (mean interpolated clone sizes 1.41 m^2^ and 0.71 m^2^ for disturbed and undisturbed forest, respectively, see Table 2).

**Table 1:** *Trientalis europaea* clonal diversity, clone sizes (log transformed), and shoot density (log transformed) with respect to minimum, interpolated size of the central clone, the minimum size of the largest clone of the patch and a mean clone size of all clones in a patch as a function of gap age class (undisturbed, <15 years, 15–60 years, and >60 years) and as contrast between undisturbed and disturbed forest stands. The tests are based on mixed models with plot considered random and gap age fixed and estimated by type III SS. NumDF is defined as degrees of freedom in the numerator and DenDF as the degrees of freedom in the denominator. The significant (*P* < 0.05) variables are shown in bold font

Effect	Gap age				Undisturbed versus disturbed			
		
Variable	NumDF	DenDF	*F*	*P*	NumDF	DenDF	*F*	*P*
Clonal diversity	3	29	1.77	0.1746	1	29	2.08	0.1603
Minimum clone size central clone (m^2^)	3	29	3.29	**0.0345**	1	29	5.67	**0.0241**
Interpolated clone size central clone (m^2^)	3	29	2.40	0.0880	1	29	5.39	**0.0275**
Minimum clone size largest clone (m^2^)	3	29	2.82	0.0564	1	29	1.91	0.1774
Mean clone size per patch (m^2^)	3	29	2.77	0.0595	1	29	1.31	0.2619
Number of shoots in central clone	3	29	3.41	**0.0304**	1	29	6.82	**0.0141**
Number of shoots in interpolated central clone	3	29	2.48	0.0810	1	29	2.20	**0.0355**
Number of shoots in largest clone	3	2	3.03	**0.0454**	1	2	2.14	0.1538
Mean number of shoots of all clones per patch	3	29	3.51	**0.0275**	1	29	2.64	0.1153
Shoot density (m^–2^)	3	29	0.37	0.7769	1	29	0.50	0.4834

**Table 2:** *Calamagrostis villosa* clonal diversity, clone sizes (log transformed), and shoot density (log transformed) with respect to minimum, interpolated size of the central clone, the minimum size of the largest clone of the patch and a mean clone size of all clones in a patch as a function of gap age class (undisturbed, <15 years, 15–60 years, and >60 years) and as contrast between undisturbed and disturbed forest stands. The tests are based on mixed models with plot considered random and gap age fixed and estimated by type III SS. NumDF is defined as degrees of freedom in the numerator and DenDF as the degrees of freedom in the denominator. The significant (*P* < 0.05) variables are shown in bold font

Effect	Gap age				Undisturbed versus disturbed			
		
Variable	NumDF	DenDF	*F*	*P*	NumDF	DenDF	*F*	*P*
Clonal diversity	3	33	1.60	0.2085	1	33	0.79	0.3794
Minimum clone size central clone (m^2^)	3	33	1.34	0.2766	1	33	0.71	0.4051
Interpolated clone size central clone (m^2^)	3	33	0.96	0.4252	1	33	2.70	0.1102
Minimum clone size largest clone (m^2^)	3	33	1.25	0.3065	1	33	1.53	0.2248
Mean clone size per patch (m^2^)	3	33	0.68	0.5691	1	33	0.90	0.3502
Number of shoots in central clone	3	33	1.30	0.2902	1	33	1.01	0.3217
Number of shoots in interpolated central clone	3	33	1.92	0.1460	1	33	5.56	**0.0244**
Number of shoots in largest clone	3	33	0.77	0.5212	1	33	1.13	0.2963
Mean number of shoots of all clones per patch	3	33	0.65	0.5898	1	33	0.95	0.3362
Shoot density (m^–2^)	3	33	0.60	0.6208	1	33	1.24	0.2739

**Figure 4:**
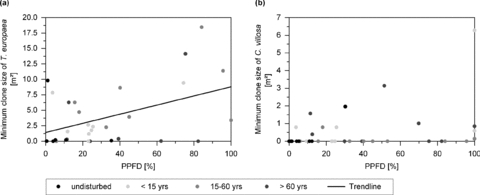
Relationship between minimum clone size of the central clone and relative light availability of (a) *T. europaea* (*r*^2^ = 0.208, *P* = 0.011, *n* = 30) and (b) *C. villosa* (*r*^2^ = 0.046, *P* = 0.052, *n* = 37).

Similar to dependence on gap age, we encountered increasing central clone sizes with increasing light availability (Fig. 4), with a significant relationship for *T. europaea* (*P* = 0.011, Fig. 4A) and a marginal one for *C. villosa* (*P* = 0.052, Fig. 4B).

The null models based on random walks gave estimated mean clone ages of the central clone that differed both between target species and gap age classes. In general, *C. villosa* had a lower mean growth distance than *T. europaea*. The estimated average expansion rate was lowest in undisturbed forest stand and highest in the youngest gaps for both species with 4.8 cm yr^–1^ and 2 cm yr^–1^ in the undisturbed forest and 38.9 cm yr^–1^ and 20.5 yr^–1^ cm in the youngest gap age class for *T. europaea* and *C. villosa*, respectively (Table 3). Under the assumption of a fixed mean annual growth rate in all gap age classes for *T. europaea* of 17.2 cm yr^–1^ after Piqueras and Klimeš (1998), we estimated a clone age of 71 years in gaps of intermediate age (15–60 years).

**Table 3:** The observed clone sizes of the central clone and the estimated expansion rate of *T. europaea* and *C. villosa* related to estimated stand ages according to gap age classes, as well as the estimated clone age for *T. europaea* in respect to a constant expansion rate

		*T. europaea*			*C. villosa*	
			
	Mean gap age (Years)	Observed mean clone sizes (m^2^)	Estimated expansion rate (cm yr^–1^)	Estimated clone age (Years)	Observed mean clone size (m^2^)	Estimated expansion rate (cm yr^–1^)
Undisturbed	200^1^	1.39	4.8	15	0.25	2
<15 years	7	3.35	38.9	36	0.94	20.5
15–60 years	35	6.56	24.3	71	0.09	3
>60 years	80	3.33	11.5	35	0.70	5.3

1 the age of 200 years was chosen arbitrarily to allow for estimating expansion rates.

**Figure 5:**
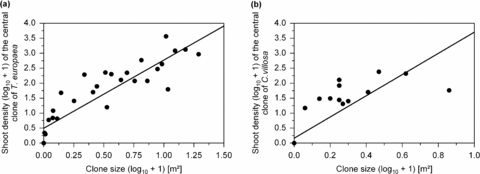
The relationship between shoot density (log_10_+ 1 transformed) and clone size (log_10_+1 transformed) of the central clone of (a) *T. europaea* (*r*^2^ = 0.802, *P* < 0.001, *n* = 33) and (b) *C. villosa* (*r*^2^ = 0.730, *P* < 0.001, *n* = 37).

**Figure 6:**
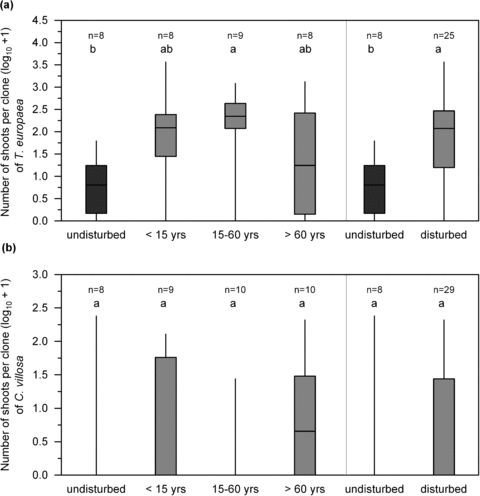
Boxplots (with median, upper and lower quartile, maximum and minimum) of the number of shoots (log_10_+1 transformed) of the central clone of (A) *T. europaea* and (B) *C. villosa* in the four gap age classes (undisturbed, <15 years, 15–60 years, and >60 years). The right-hand side of the graph shows the contrast between undisturbed versus gap plots. Small letters indicate statistically significant differences between the gap age classes according to the Tukey–Kramer post-hoc test.

Furthermore, shoot density increased with clone size (Fig. 5), with a significant relationship for both species (*P* < 0.001). For *T. europaea*, the pattern of shoot density of the central clone among gap age classes (Fig. 6A) was very similar to the pattern of patch sizes (Fig. 3A). The density of the central clone was highest in gaps between 15 and 60 years and lowest in undisturbed forest. However, no difference in shoot density was encountered for *C. villosa* (Fig. 6B), which was in accordance to the results on clone size.

### Genetic population structure and genetic diversity

In general, the clonal richness was significantly higher for *C. villosa* (*R* = 0.55) than for *T. europaea* (*R* = 0.47, *F* = 7.48, *P* = 0.011). The amount of genetic variation was higher within populations than between populations for both *T. europaea* and *C. villosa*, whereas the gap age classes did not contribute at all to genetic variation (Table 4). This pattern was even clearer in the genet dataset with 86% of the variance encountered within population and only 14% between the populations for *T. europaea*. The same pattern with somewhat lower amounts of variation was found for *C. villosa*. Accordingly, the Φ_PT_ value was larger for ramets than for genets and higher for *C. villosa* than for *T. europaea* (Table 4).

**Table 4:** Analyses of molecular variance (AMOVA) of *T. europaea* and *C. villosa*. The Φ-values among gap age classes and populations are Φ_RT_ and Φ_PT_, respectively

	*T. europaea*			*C. villosa*		
		
Replicates/source	DF	Percent of total variance	Φ-value	DF	Percent of total variance	Φ-value
Ramets						
Among gap age classes	3	0	0.002	3	0	0.004
Among populations	39	29	0.293	39	39	0.392
Within populations	489	71		531	61	
Genets						
Among gap age classes	3	0	0.002	3	0	0.004
Among populations	36	14	0.141	39	28	0.278
Within populations	209	86		272	72	

**Figure A2:**
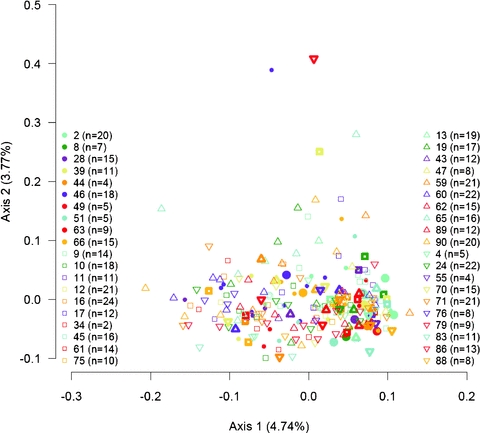
The PCoA of the genets of *T. europaea*.

**Figure A3:**
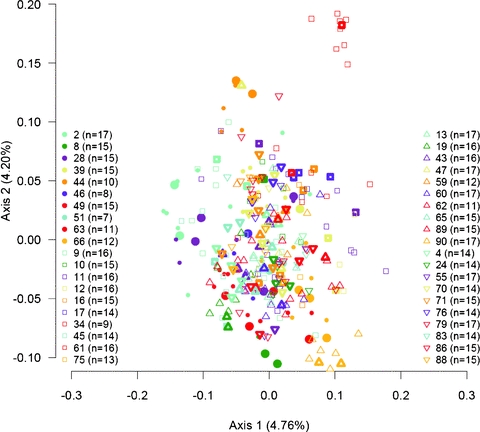
The PCoA of the genets of *C. villosa*.

**Figure A4:**
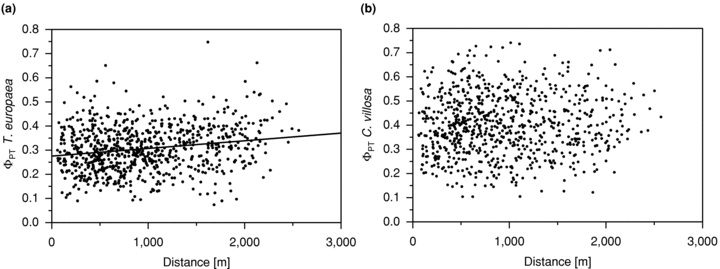
The Mantel-test of the pairwise Φ_PT_ values per population of the two target species (a) *T. europaea* (*r*^2^ = 0.037, *P* = 0.004) and (b) *C. villosa* (*r*^2^ = 0.003, *P* = 0.241).

The high variation within and the low variation between the populations was also reflected in the PCoAs of the two species (Figs. A2 and A3), revealing neither clear groups of age classes nor populations in both species. *Trientalis europaea* showed a clear isolation by distance (*P* = 0.004, Fig. A4), a pattern not encountered for *C. villosa* (*P* = 0.241, Fig. A4).

## Discussion

Using AFLP for molecular fingerprinting, we could demonstrate that patch and clone sizes depended on gap age. Thus, the approach was able to capture an important ecological process, that is, herb species regeneration in canopy gaps, that otherwise has not yet been amenable to direct observation.

Our first hypothesis was principally confirmed, at least for one of our target species, *T. europaea*. Although we failed to show a unimodal relationship between clone size and gap age for *C. villosa*, we demonstrated this unimodal relationship for the patch size of *C. villosa*. Moreover, we could demonstrate that the underlying factor driving clonal growth probably was light availability. Vegetative growth of *T. europaea* seems to be favored in early stages of gap development, while subsequently decreasing light availability seems to hamper clone expansion. This conforms to a preceding study that showed that biomass accumulation of *T. europaea, C. villosa*, as well as of *V. myrtillus* was also positively related to light (Patsias and Bruelheide 2011). However, this biomass accumulation did not only result from clone expansion but also from higher ramet density. In particular, *V. myrtillus* increased branching intensity under improved light availability (Patsias and Bruelheide 2011).

Furthermore, our results showed that the dependence on gap age was not linear. By simulation, we calculated a clone age of 71 years of *T. europaea* in the mid-successional gaps, that is, clones had to exist already before gap creation. However, the environmental conditions in gaps are superior to those in undisturbed forest. Therefore, clone sizes of *T. europaea* increased initially after gap formation, but then leveled off during gap development, and finally will probably decrease to pregap conditions. Similarly, Moola and Vasseur (2009) described such a pattern for the understorey dwarf shrub *Gaultheria procumbens* in Canada. After clear-cutting, the number of ramets increased and achieved a maximum 8 years after clear-cutting, whereas the ramet density was as low as in the undisturbed forest 56 years after clear-cutting (Moola and Vasseur 2009). The underlying reason for this pattern probably is the weak competitive strength of *T. europaea*, as has been described also for five grassland species with far-reaching guerrilla-type ramets (Wildová et al. 2007). In the study of Wildová et al. (2007), fast colonizers achieved new gaps earlier than slow colonizers, but then finally were replaced by them. Although *C. villosa* also displays the guerrilla strategy, this species certainly has a higher competition ability, which is also reflected in the encountered independence of gap age. Similarly, the congeneric *C. canadensis* is a highly competitive clonal grass species that suppress the regeneration of *Picea glauca* (Landhäusser and Lieffers 1999; Matsushima and Chang 2007).

Our second hypothesis of a larger clone size for fast growing species compared to slow growing species was confirmed as *T. europaea* had larger clones than *C. villosa*. The high clone expansion rate of *T. europaea* is connected to the species’ pseudoannual life form, which results in a very high mobility (Økland 1995). The mother ramet dies back every year, consequentially the ramets are able to change their position from year to year without being dependent on static mother ramets, as this is the case for newly created ramets of *C. villosa*. Under favorable conditions, shoots of *T. europaea* can cover distance of 1 m from the previous year's mother ramet (Piqueras and Klimeš 1998). Our clone sizes for the central clone of *T. europaea* are in the range of the closely related *T. borealis*, described by Whitford (1949) to range between 2.63 and 16.4 m^2^. In contrast, the rhizome length of *C. villosa* is traded off by a denser root system (Betz 1998).

Our third hypothesis of a positive relationship between ramet density and clone size was also confirmed. These results are consistent with the findings of Flower–Ellis (1971), who described positive relationships between clone size and age as well as for ramet density and clone age for *V. myrtillus*. There are also other species, for instance *Paris quadrifolia* (herb), *Ilex leucoclada* (shrub), or *Picea mariana* (tree), for which both patch expansion and increasing “filling-in” of ramets inside patches has been described for increasing clone sizes (Laberge et al. 2000; Jacquemyn et al. 2005; Torimaru and Tomaru 2005).

The gap age class did not have any impact on the population structure of *T. europaea* and *C. villosa*. The high amount of genetic variation encountered within populations showed that both species were not exclusively dependent on vegetative reproduction. This is consistent with our findings that most of the investigated patches (96%) contain more than one clone. The high clonal richness that was independent from gap age hints at a low competition intensity between different genets of a patch. The combination of reproduction modes seem to be the rule rather than an exception, as seen from Eriksson's (1989) review, where 40% of the investigated clonal species (*n* = 68) also displayed sexual reproduction. Accordingly, RAPD analyses of *V. stamineum* in South Carolina revealed that 91% of the investigated patches contained more than one clone (Kreher et al. 2000). The frequent incidence of sexual reproduction is also reflected in a high molecular variance within compared to among populations. For the congeneric species *V. vitis-idaea*, Persson and Gustavsson (2001) described 89.2% and 10.8% for the molecular variance within and among populations, respectively. *Calamagrostis epigejos*, a species with tremendous increases in conifer forests in the last decades (Zerbe and Brande 2003) and related to *C. villosa*, showed a similar population structure, reflected in comparably low Φ_PT_ values (Lehmann 1997).

The somewhat higher Φ_PT_ values of *C. villosa* compared to *T. europaea* point to a lower gene flow for *C. villosa*. Using Wright's (1931) formula for the “infinite island model, ” the number of migrants *Nm* are 1.52 and 0.65 for *T. europaea* and *C. villosa*, respectively. This conclusion is supported by our findings on the relationships between genetic and geographic distances, as a significant isolation by distance was only encountered for *T. europaea*, showing that an equilibrium between gene flow and genetic drift can only be assumed for this species (Hutchison and Templeton 1999).

In conclusion, we showed that the colonization pattern of herb layer species after canopy disturbance in this spruce forest depended on vegetative growth form (guerrilla or phalanx type). Moreover, species of the same strategy type (i.e., guerrilla strategy of *T. europaea* and *C. villosa*) differed in colonization pattern and in genetic diversity. A distinct relationship to gap age with maximum clone sizes in mid-successional stages was only encountered for the highly mobile herb *T. europaea*. This finding offers interesting perspectives for dating ecological processes, as species such as *T. europaea* can be used as “ecological clock, ” estimating the time elapsed since the onset of the process.

## Appendix

**Table A1:** Number of clones per patch, as revealed by AFLP analyses, minimum clone size of the central clone per patch, the mean minimum clone size of the central clone, as well as the interpolated clone sizes and the mean interpolated clone sizes per gap age class for *T. europaea* and *C. villosa*

	*T. europaea*					*C. villosa*				
		
Gap age	Number of clones	Minimum clone size (central clone) (m^2^)	Mean minumum clone size (central clone) ± standard deviation (m^2^)	Interpolated clone size (central clone) (m^2^)	Mean interpolated clone size (central clone) ± standard deviation (m^2^)	Number of clones	Minimum clone size (central clone) (m^2^)	Mean minumum clone size (central clone) ± standard deviation (m^2^)	Interpolated clone size (central clone) (m^2^)	Mean interpolated clone size (central clone) ± standard deviation (m^2^)
Undisturbed	12	0.00	1.39 ± 3.42	0.79	3.28 ± 6.71	13	0.00	0.25 ± 0.69	0.79	0.71 ± 1.20
	3	0.19		0.25		3	0.00		0.19	
	4	9.82		19.73		9	0.00		0.29	
	5	0.20		2.60		9	0.00		0.29	
	1	0.02		0.02		7	0.00		0.34	
	9	0.00		0.44		3	0.00		0.02	
	NA	NA		NA		8	1.96		3.63	
	NA	NA		NA		NA	NA		NA	
	4	0.10		0.44		5	0.00		0.12	
	8	0.79		1.96		NA	NA		NA	
15 yrs	12	0.00	3.35 ± 3.39	1.96	8.66 ± 9.15	13	0.00	0.94 ± 2.04	0.79	1.63 ± 2.16
	10	2.65		2.97		7	0.00		0.29	
	6	1.77		2.01		14	0.00		0.20	
	7	9.42		27.88		NA	NA		NA	
	11	7.85		16.30		8	0.79		2.55	
	7	2.36		7.66		8	0.79		2.95	
	NA	NA		NA		3	0.59		0.79	
	7	1.18		2.75		12	0.00		0.20	
	10	1.57		7.76		14	0.00		0.20	
	NA	NA		NA		3	6.28		6.68	
15-60 yrs	7	11.39	6.56 ± 5.60	18.46	10.22 ± 8.22	8	0.00	0.09 ± 0.25	0.79	1.13 ± 0.84
	8	0.00		0.38		9	0.00		0.38	
	2	4.71		4.71		8	0.79		2.75	
	NA	NA		NA		4	0.00		0.79	
	8	8.64		12.66		7	0.00		2.36	
	10	6.28		14.53		13	0.00		0.79	
	5	2.26		3.49		9	0.00		0.20	
	6	18.46		25.72		8	0.00		0.98	
	7	3.93		6.19		12	0.00		1.57	
	8	3.39		5.80		12	0.14		0.71	
60 yrs	NA	NA	3.33 ± 5.09	NA	7.83 ± 10.07	6	3.14	0.70 ± 1.02	5.40	1.49 ± 1.52
	8	14.14		29.26		7	0.00		0.59	
	NA	NA		NA		5	0.85		0.99	
	6	6.28		11.78		7	0.39		1.57	
	11	5.50		9.72		7	0.00		2.16	
	5	0.00		0.67		6	0.00		0.32	
	4	0.00		0.05		8	0.00		0.20	
	5	0.29		0.59		5	1.57		1.82	
	9	0.39		10.50		10	0.00		0.59	
	4	0.03		0.04		5	1.01		1.27	
